# Tools and algorithms for the construction and analysis of systems: a special issue for TACAS 2020

**DOI:** 10.1007/s10009-022-00677-9

**Published:** 2022-09-23

**Authors:** Armin Biere, David Parker

**Affiliations:** 1 Albert-Ludwigs University, Freiburg, Germany; 2 School of Computer Science, University of Birmingham, Birmingham, UK

**Keywords:** Software verification, Theorem proving, Model checking, Static analysis, Runtime verification, Probabilistic verification, Compositional verification, Symbolic execution, Concurrent systems, Formal specification techniques, SAT and SMT solving, Tool environments and tool architectures, Software engineering and maintenance

## Abstract

This special issue of Software Tools for Technology Transfer comprises extended versions of selected papers from the 26th edition of the International Conference on Tools and Algorithms for the Construction and Analysis of Systems (TACAS 2020). The focus of this conference series is tools and algorithms for the rigorous analysis of software and hardware systems, and the papers in this special cover the spectrum of current work in this field.

## TACAS 2020

The International Conference on Tools and Algorithms for the Construction and Analysis of Systems (TACAS) is a forum for researchers and practitioners working with formal tools and techniques for building and analyzing computerized systems. It aims to bridge the gaps between different communities with this common interest and to support them in their quest to improve the utility, reliability, flexibility, and efficiency of tools and algorithms for building systems.

TACAS 2020 [1, 2] was the 26th edition of this conference, forming part of the 23rd European Joint Conferences on Theory and Practice of Software (ETAPS 2020). Originally planned to be held in Dublin, Ireland, in April 2020, the event itself was unfortunately cancelled due to the global COVID-19 pandemic. However, TACAS 2020 still attracted an impressive selection of high-quality papers covering the full array of ongoing work in this field, and authors later had the chance to present their work at the following year’s iteration of the conference.

## This special issue

TACAS 2020 [1, 2] received 155 paper submissions, of which 48 were ultimately accepted. Paper selection was performed by a program committee of 35 members, with each paper receiving at least three reviews. Based on the scores from reviewers, and after considering the papers and submitted reviews, the program chairs invited the authors of the top-ranked 13 full-length papers to submit extended versions to this special issue. Ultimately, 9 papers were accepted, each having received at least two reviews. They are included in this issue, and are briefly summarized below.

### Assume, guarantee or repair: a regular framework for non regular properties

In paper [3], Hadar Frenkel, Orna Grumberg, Corina S. Păsăreanu and Sarai Sheinvald present a framework that both verifies a concurrent program and repairs it in the case that verification fails. The work takes a compositional approach, building on the classic assume-guarantee style of reasoning, incorporating a learning-based method to find assumptions and adding an automated, incremental approach to repair the program if required. Results from an implementation demonstrate its applicability to a range of concurrent program benchmarks.

### A calculus for modular loop acceleration and non-termination proofs

In paper [4], Florian Frohn and Carsten Fuhs present a novel approach for loop acceleration, a set of techniques that underlies static analysis methods for checking a variety of correctness properties of programs. The paper presents a calculus designed to allow multiple acceleration techniques to be combined in a modular fashion. Furthermore, two new loop acceleration methods are included, and an empirical evaluation of the overall framework contrasts its performance with related techniques.

### The CoLiS platform for the analysis of maintainer scripts in Debian software packages

In paper [5], Benedikt Becker, Nicolas Jeannerod, Claude Marché, Yann Régis-Gianas, Mihaela Sighireanu and Ralf Treinen describe a software platform for checking maintainer scripts in the Debian distribution against compliance to policies regarding various technical aspects. The overall methodology is based on two techniques: symbolic execution and feature tree constraints. The paper describes the approach and its deployment on existing Debian scripts, highlighting policy violations in a significant number of cases.

### Partial-order reduction for parity games and parameterized Boolean equation systems

In paper [6], Thomas Neele, Tim A.C. Willemse, Wieger Wesselink and Antti Valmari study partial order reduction, a well known technique for tackling the state space explosion problem in the context of model checking parallel programs. They present novel partial order reduction methods for verifying parity games, represented using parameterized Boolean equation systems, yielding an approach that is sound for the full modal $\mu$-calculus. An implementation is evaluated and shown to provide good reductions on a range of benchmark programs.

### The Discourje project: runtime verification of communication protocols in Clojure

In paper [7], Ruben Hamers, Erik Horlings and Sung-Shik Jongmans provide a broad overview of a project that aims to provide runtime verification support for concurrent programs written in Clojure, a dynamically typed, functional language which compiles to Java bytecode. The Discourje project provides support for instrumentation and construction of monitors, in particular for for concurrent programs that use channels, and dynamic analysis methods to catch concurrency bugs.

### Verifying OpenJDK’s linkedList using KeY

In paper [8], Hans-Dieter A. Hiep, Olaf Maathuis, Jinting Bian, Frank S. de Boer and Stijn de Gouw present a real-world case study of software verification, targeting an implementation of the linked list data structure in the Java Collection Framework. The paper describes the process of formally specifying and verifying the LinkedList class, using the KeY theorem prover, including the discovery of and a fix for an integer overflow bug.

### Scenario-based verification of uncertain parametric MDPs

In paper [9], Thom Badings, Murat Cubuktepe, Nils Jansen, Sebastian Junges, Joost-Pieter Katoen and Ufuk Topcu present techniques to verify probabilistic temporal logic specifications against uncertain parametric Markov decision processes. This is a probabilistic model for sequential decision-making problems in which some transitions are described by unknown probability distributions over parameter values. The authors propose a sampling-based approach to generate tight and high-confidence bounds on the probability of satisfying a specification, evaluated on a range of standard benchmarks.

### Analysis of non-Markovian repairable fault trees through rare event simulation

In paper [10], Carlos E. Budde, Pedro R. D’Argenio, Raúl E. Monti and Mariëlle Stoelinga present formal methods for the analysis of dynamic fault trees, a formalism for assessing the dependability of safety-critical systems used in the field of reliability engineering. They propose Monte Carlo simulation techniques, focusing in particular on an automated approach to discovering so-called importance functions, which can be used to effectively analyze systems where the events of interest occur only very rarely. Experimental results illustrate the techniques on a selection of case studies.

### Full-program induction: verifying array programs sans loop invariants

In paper [11], Supratik Chakraborty, Ashutosh Gupta and Divyesh Unadkat propose techniques for verifying the safety of programs that use arrays. The approach is called full-program induction and targets programs that manipulate arrays of parametric size, using fully automated program transformations that avoid the need for generation of loop invariants. The performance of a prototype tool implementing this idea is evaluated on a set of software verification benchmarks.
